# Bone Turnover Markers as Biomarkers of Cough Dysfunction and Respiratory Risk in Subacute Ischemic Stroke

**DOI:** 10.3390/diagnostics16071008

**Published:** 2026-03-27

**Authors:** Ki-Hyeok Ku, Seung Don Yoo, Dong Hwan Kim, Seung Ah Lee, Sung Joon Chung, Jinkyeong Park, Sae Rom Kim, Eo Jin Park

**Affiliations:** 1 Department of Orthopedic Surgery, Kyung Hee University College of Medicine, Kyung Hee University Hospital at Gangdong, Seoul 05278, Republic of Korea; 2 Department of Rehabilitation Medicine, Kyung Hee University College of Medicine, Kyung Hee University Hospital at Gangdong, Seoul 05278, Republic of Korea; 3 Department of Pulmonary, Allergy and Critical Care Medicine, Kyung Hee University Hospital at Gangdong, Seoul 05278, Republic of Korea

**Keywords:** bone turnover markers, C-terminal telopeptide, peak cough flow, aspiration pneumonia, ischemic stroke

## Abstract

**Background/Objectives**: Peak cough flow (PCF) is an objective measure of cough effectiveness after stroke, but biomarkers reflecting physiological vulnerability related to reduced PCF are not well established. We investigated whether bone turnover markers (BTMs)—C-terminal telopeptide of type I collagen (CTX) and procollagen type 1 N-terminal propeptide (P1NP)—were associated with PCF in subacute ischemic stroke. **Methods**: In this retrospective study, 112 patients admitted within 21 days of stroke onset had fasting morning CTX and P1NP measured by electrochemiluminescence immunoassay, and PCF measured within 72 h of admission. Associations were assessed using Spearman correlation and multivariable linear regression with BTMs standardized (per 1 standard deviation increase), adjusting for age, sex, body mass index, onset-to-admission days, National Institutes of Health Stroke Scale score, Korean version of the Modified Barthel Index, estimated glomerular filtration rate, smoking status, and brainstem lesion. **Results**: CTX showed an inverse correlation with PCF (rho = −0.469; *p* < 0.001) and remained independently associated with lower PCF after multivariable adjustment (β = −42.32 L/min; 95% confidence interval, −56.12 to −28.52; *p* < 0.001), whereas P1NP showed weaker associations. In secondary outcome analyses, higher CTX was associated with low PCF (PCF < 160 L/min), aspiration pneumonia, and longer length of stay. **Conclusions**: Higher CTX levels were independently associated with lower peak cough flow and selected respiratory-related outcomes in this retrospective cohort. These findings are hypothesis-generating, do not imply prognostic validation, and warrant confirmation in prospective multicenter studies assessing incremental predictive value.

## 1. Introduction

Ischemic stroke frequently impairs airway protection and effective coughing, creating a high-risk window for respiratory complications that may hinder neurological recovery and prolong hospitalization [1,2]. In addition to dysphagia, a weak cough limits the clearance of secretions and aspirated debris, increasing the risk of aspiration pneumonia and atelectasis and often necessitating more intensive airway management [1,3]. Given the prevalence and clinical significance of pulmonary complications after stroke, practical methods to identify patients with increased respiratory vulnerability are essential during early inpatient care and rehabilitation [4,5,6].

Peak cough flow (PCF) is an objective bedside measure of cough effectiveness [7,8]. An effective cough requires strong expiratory muscle contraction, coordinated glottic closure, and adequate inspiratory effort to generate high airflow for airway clearance [8,9]. These mechanisms may be compromised after stroke through several pathways. Neurological impairment can reduce recruitment of trunk and abdominal muscles, disrupt central coordination of respiratory muscles, and impair bulbar function supporting airway reflexes [10,11,12]. In addition, immobility, fatigue, and malnutrition can accelerate respiratory and trunk muscle weakness in patients with stroke [11,13]. Clinically, ineffective coughing can lead to secretion retention, frequent suctioning, and increased susceptibility to aspiration-related pulmonary complications [1,3].

Neurological severity scales and lesion characteristics do not fully predict the risk of post-stroke respiratory complications [14,15,16]. In combination with immobilization and reduced nutritional intake, acute stroke can trigger inflammatory and neuroendocrine responses that shift the body toward a catabolic state [13,17,18]. Skeletal muscle atrophy after acute stroke may result from this systemic catabolic response, leading to lean mass loss and activation of catabolic pathways; concurrently, early post-stroke immobility is associated with increased bone resorption and rapid bone loss, which may further complicate rehabilitation [19,20,21,22]. Accordingly, biomarkers reflecting systemic metabolic stress may complement clinical assessments and provide additional insight into individual differences in respiratory vulnerability [23,24].

Bone turnover markers (BTMs) provide insight into skeletal remodeling and, more broadly, into metabolic and inflammatory states that affect musculoskeletal integrity [25,26]. Procollagen type 1 N-terminal propeptide (P1NP), which reflects bone formation, and C-terminal telopeptide of type I collagen (CTX), which reflects bone resorption, are two commonly used markers [25,27]. Bone remodeling may shift toward increased resorption and altered coupling of formation and resorption in settings of inactivity, inflammation, and acute illness [28,29,30]. Accordingly, BTMs may capture a systemic vulnerability phenotype rather than solely skeletal changes, as signals related to bone remodeling have been linked to frailty, muscle function, and functional decline across diverse clinical contexts [31,32,33].

From this perspective, increased bone resorption, reflected by elevated CTX, may represent or accompany a catabolic state in which trunk and respiratory muscles are also affected [31,34,35]. During the early recovery phase, this phenotype may manifest as reduced cough flow and increased demands for airway care [8,36,37]. In contrast, during acute illness and recovery, bone formation markers such as P1NP may demonstrate more complex and time-dependent responses, potentially leading to greater variability in their associations with early functional measures [38,39]. Despite this biological rationale, the relationship between cough efficacy and BTMs in stroke populations remains poorly characterized [8,26,37].

Accordingly, this study examines the association between BTMs (CTX and P1NP) and cough function, as assessed by PCF, in patients with ischemic stroke during the early recovery period. This study evaluates biomarker associations rather than validated clinical utility; therefore, the findings should be interpreted as hypothesis-generating and require prospective validation before clinical implementation. We further explore whether BTMs are associated with clinically relevant respiratory-related outcomes, including inadequate cough thresholds, aspiration-related complications, length of hospitalization, and requirements for secretion management, which reflect airway vulnerability and inpatient burden. We hypothesize that lower PCF and worse respiratory-related outcomes are associated with higher BTM levels. By linking bone-remodeling biomarkers to an objective measure of cough effectiveness, this work seeks to expand the biomarker framework for respiratory risk assessment in stroke care and generate hypotheses for future longitudinal and interventional studies.

## 2. Materials and Methods

### 2.1. Subjects

This retrospective single-center observational study was conducted at Kyung Hee University Hospital at Gangdong. Consecutive patients admitted to the Department of Rehabilitation Medicine between January 2020 and December 2024 were screened. Eligible participants were adults (≥19 years) with ischemic stroke confirmed by neuroimaging who were admitted during the subacute phase, defined as an onset-to-admission interval of ≤21 days. Patients were included if measurements of BTMs—CTX and P1NP—and PCF were available during the index hospitalization.

Patients were excluded if key variables for the primary analysis (CTX, P1NP, or PCF) were missing. Additional exclusion criteria were as follows: conditions expected to substantially affect cough performance independent of ischemic stroke, such as prior intubation or tracheostomy, vocal cord paralysis or other laryngeal lesions, chronic lung disease (e.g., chronic obstructive pulmonary disease or interstitial lung disease), severely impaired consciousness, and inability to perform PCF testing due to severe aphasia or cognitive impairment [40,41,42,43,44]; and conditions and treatments expected to substantially influence bone turnover, such as advanced chronic kidney disease (estimated glomerular filtration rate (eGFR) <30 mL/min/1.73 m^2^) or dialysis, malignancy, recent major fracture or orthopedic surgery (within 3 months), chronic systemic corticosteroid use (e.g., prednisone ≥ 5 mg/day for ≥3 months), use of osteoporosis medications (bisphosphonates, denosumab, or teriparatide), major endocrine disorders affecting bone metabolism (e.g., hyperparathyroidism or hyperthyroidism), and non-ischemic intracranial lesions (e.g., hemorrhagic stroke or brain tumor) [40,41,42,43,44]. For covariates, analyses were performed using available cases for each model (complete-case per model), and the analyzed sample size (N) is reported for each model/table.

### 2.2. Measurements and Variables

Demographic and clinical data were extracted from electronic medical records, including age, sex, body mass index (BMI), onset-to-admission interval, stroke severity assessed by the National Institutes of Health Stroke Scale (NIHSS), functional status measured by the Korean version of the Modified Barthel Index (K-MBI), kidney function estimated by eGFR (mL/min/1.73 m^2^), and smoking status.

BTMs (serum CTX and P1NP) were measured using electrochemiluminescence immunoassays in the hospital’s central laboratory according to the manufacturer’s instructions. Blood samples were obtained within 72 h of admission, collected in the morning after an overnight fast, and results were reported in ng/mL.

PCF (L/min) was assessed within 72 h of admission using a handheld peak flow meter (Philips Respironics Inc., Murrysville, PA, USA) by a trained therapist with the patient in a seated position. Participants were instructed to take a deep breath and then cough as forcefully as possible into the device [45,46]. The test was repeated three times with brief rest periods, and the highest value was recorded for analysis [45,46]. BTM sampling and PCF assessment were both performed within 72 h of admission. PCF assessment was conducted in routine workflow by trained therapists and was not contingent on BTM results.

The primary outcome was PCF measured as a continuous variable (L/min). Prespecified secondary outcomes included low PCF, aspiration pneumonia during hospitalization, length of stay (LOS), and suction burden. Low PCF was defined as PCF < 160 L/min [8,36]. Aspiration pneumonia during hospitalization was defined in this study as the presence of (1) new or progressive pulmonary infiltrates on chest imaging (chest radiography or computed tomography) and (2) initiation of systemic antibiotic therapy for suspected pneumonia [47,48]. LOS was defined as the number of inpatient days from the date of admission to the date of discharge. Suction count was defined as the total number of documented airway suctioning events recorded in nursing charts during the index hospitalization [49,50].

### 2.3. Statistical Analysis

Shapiro–Wilk tests indicated departures from normality for CTX, P1NP, and PCF; therefore, unadjusted associations between each BTM and PCF were assessed using Spearman’s rank correlation (rho). For adjusted analyses, we used multivariable linear regression with PCF as the dependent variable because the primary objective was to estimate mean differences in PCF associated with each biomarker after covariate adjustment, and residual diagnostics did not indicate substantial violation of model assumptions. CTX and P1NP were modeled in separate regression models and standardized as z scores to facilitate interpretation per 1 standard deviation (SD) increase and comparison across markers measured on different scales; effect estimates are reported as β coefficients with 95% confidence intervals (CIs) and *p* values. Prespecified covariates included age, sex, body mass index (BMI), onset-to-admission interval, NIHSS, K-MBI, eGFR, smoking status (smoker vs. non-smoker), and brainstem lesion status. Covariates were selected a priori based on clinical relevance and potential confounding. Model fit was summarized using adjusted R^2^.

For secondary outcomes, parsimoniously adjusted models were used to mitigate overfitting given limited event counts: logistic regression for binary outcomes, log-transformed linear regression for right-skewed LOS, and negative binomial regression for overdispersed count outcomes (suction count). Low PCF (PCF < 160 L/min) and aspiration pneumonia were analyzed using logistic regression, with results reported as odds ratios (ORs) with 95% CIs and *p* values per 1 SD increase in CTX or P1NP. LOS was analyzed using linear regression with log-transformed LOS to account for right skewness; estimates were back-transformed and reported as percent changes in LOS per 1 SD increase in CTX or P1NP. Suction count was analyzed as a count outcome using negative binomial regression, and results are reported as incidence rate ratios (IRRs) with 95% CIs and *p* values. All statistical analyses were performed using IBM SPSS Statistics for Windows, version 20.0 (IBM Corp., Armonk, NY, USA). Multicollinearity was assessed using variance inflation factors (VIFs) and tolerance. All VIFs were <1.14 (maximum VIF = 1.13), including for NIHSS and K-MBI. For linear regression, residual plots and standardized residual distributions were inspected to assess assumptions; there was no evidence of substantial non-normality (Shapiro–Wilk *p* = 0.114) or heteroscedasticity (Breusch–Pagan *p* = 0.374). For logistic regression models, goodness-of-fit was assessed using the Hosmer–Lemeshow test (low PCF model *p* = 0.018; pneumonia model *p* = 0.908). The Hosmer–Lemeshow test can be sensitive to the number of risk groups, particularly in modest sample sizes; therefore, calibration results were interpreted cautiously, and the secondary models were not intended as prognostic tools. As a sensitivity analysis for the primary CTX–PCF association, we refitted the multivariable CTX model after excluding influential observations defined by Cook’s distance > 4/*n* (*n* = 6); the CTX effect remained materially unchanged.

## 3. Results

### 3.1. Study Population and Baseline Characteristics

Baseline characteristics were summarized to describe the cohort and provide context for interpreting subsequent associations between BTMs and PCF. A total of 112 patients with subacute ischemic stroke were included in the analyses (Table 1). The mean age was 63.7 ± 14.1 years, and 61.6% were male. The mean onset-to-admission interval was 13.8 ± 4.4 days. Mean NIHSS and K-MBI scores were 13.7 ± 6.5 and 49.4 ± 24.1, respectively. The mean PCF was 237.0 ± 85.2 L/min. Mean CTX and P1NP levels were 0.55 ± 0.26 and 54.25 ± 23.56 ng/mL, respectively. Low PCF (PCF < 160 L/min) was observed in 25 (22.3%) patients, and aspiration pneumonia occurred in 26 (23.2%). Brainstem lesions were present in 30 (26.8%) patients, and 39 (34.8%) patients were current smokers (Table 1).

### 3.2. Correlation Between BTM and PCF

**Figure 1:**
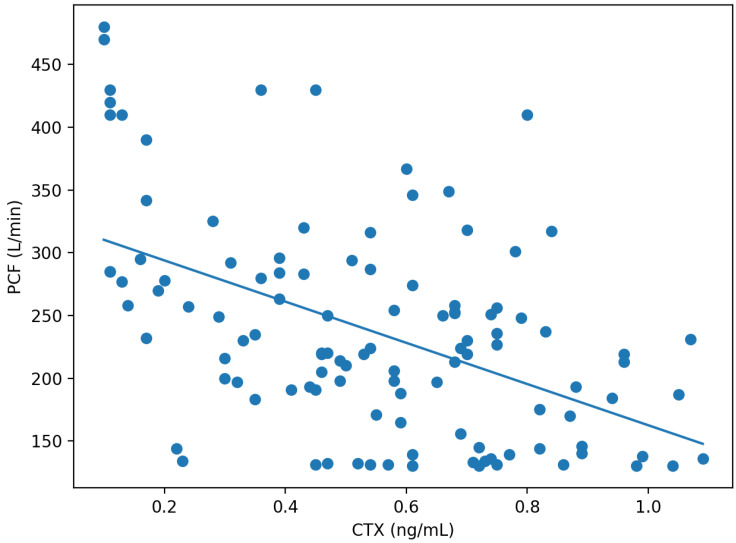
Scatterplot of serum CTX and peak cough flow (PCF) in subacute ischemic stroke patients. Each point represents one participant. The solid line indicates the fitted linear regression line.

We first examined unadjusted correlations to identify potential associations between BTMs and PCF before accounting for confounders. Shapiro–Wilk testing indicated departures from normality for CTX (*p* = 0.032), P1NP (*p* = 0.019), and PCF (*p* < 0.001). In Spearman correlation analyses, CTX showed a moderate inverse correlation with PCF (rho = −0.469, *p* < 0.001), whereas P1NP showed a weaker inverse correlation with PCF (rho = −0.213, *p* = 0.024) (Table 2, Figure 1). In partial Spearman correlation adjusting for NIHSS, CTX remained inversely associated with PCF (partial rho = −0.494, *p* < 0.001).

### 3.3. Multivariable Linear Regression for PCF

We then fitted multivariable linear regression models to estimate adjusted associations between each BTM and PCF while controlling for prespecified covariates. In multivariable linear regression models adjusting for prespecified covariates (age, sex, BMI, onset-to-admission interval, NIHSS, K-MBI, eGFR, smoking status, and brainstem lesion), higher CTX remained independently associated with lower PCF. Specifically, per 1 SD increase in CTX, PCF decreased by 42.32 L/min (β = −42.32, 95% CI −56.12 to −28.52, *p* < 0.001). The CTX model explained a moderate proportion of the variance in PCF (adjusted R^2^ = 0.294) (Table 3).

In the corresponding model for P1NP, P1NP was also inversely associated with PCF, although with a smaller effect size: per 1 SD increase in P1NP, PCF decreased by 18.33 L/min (β = −18.33, 95% CI −34.28 to −2.38, *p* = 0.025). The overall model fit was lower (adjusted R^2^ = 0.082) (Table 3).

Across both models, higher NIHSS scores were consistently associated with lower PCF (CTX model, β = −3.27, 95% CI −5.40 to −1.13, *p* = 0.003; P1NP model, β = −2.48, 95% CI −4.94 to −0.03, *p* = 0.048). Other covariates, including K-MBI, eGFR, smoking status, and brainstem lesion, were not significantly associated with PCF in these adjusted models (Table 3). In sensitivity analysis excluding influential observations (Cook’s distance > 4/n; n = 6), the association remained similar (β = −37.29 L/min; 95% CI −49.54 to −25.04; *p* < 0.001).

### 3.4. Secondary Outcomes

Finally, we explored clinically relevant respiratory-related outcomes using parsimoniously adjusted models to mitigate overfitting given limited event counts. In parsimoniously adjusted models for secondary outcomes (events: low PCF = 25, pneumonia = 26), CTX was associated with a higher likelihood of low PCF; per 1 SD increase in CTX, the odds of low PCF increased nearly threefold (OR 2.93, 95% CI 1.59–5.40, *p* < 0.001). CTX was also associated with aspiration pneumonia (OR 1.70, 95% CI 1.02–2.81, *p* = 0.040) (Table 4). However, the pneumonia estimate had borderline precision (95% CI 1.02–2.81) and should be interpreted cautiously.

With respect to the hospitalization course, higher CTX was associated with longer LOS after log transformation, corresponding to an estimated 9.0% increase in LOS per 1 SD increase in CTX (95% CI 1.7–16.9%, *p* = 0.017). CTX was not significantly associated with suction burden (IRR 1.17, 95% CI 0.97–1.42, *p* = 0.104) (Table 4). Suction counts may be influenced by practice patterns and documentation variability.

In contrast, P1NP was not significantly associated with low PCF (OR 1.47, *p* = 0.133), aspiration pneumonia (OR 1.04, *p* = 0.859), LOS (0.3%, *p* = 0.946), or suction count (IRR 0.96, *p* = 0.649) in the adjusted secondary analyses (Table 4).

## 4. Discussion

To the best of our knowledge, this is one of the first studies to examine the relationship between cough strength, measured by PCF, and BTMs in patients with subacute ischemic stroke. In both correlation and multivariable regression analyses, higher CTX was consistently associated with reduced PCF, whereas the relationship between P1NP and PCF was weaker and less consistent. In addition, CTX was associated with longer LOS and clinically relevant respiratory-related outcomes, such as low PCF and aspiration pneumonia, in exploratory secondary analyses using parsimonious adjustment to reduce the risk of overfitting. Although these findings do not establish causality or a validated prediction model, they indicate that CTX may reflect early physiological susceptibility relevant to cough effectiveness during the subacute phase after stroke.

An effective cough requires strong expiratory muscle contraction, glottic control, and coordinated inspiratory effort to generate high expiratory airflow for airway clearance [51,52]. Following ischemic stroke, coughing may be impaired by several factors, such as disrupted central coordination of respiratory muscles, reduced activation of trunk and abdominal muscles, and bulbar dysfunction affecting airway reflexes. In addition, systemic inflammation, neuroendocrine stress responses, early post-stroke immobilization, and reduced caloric intake may accelerate deconditioning and diminish overall musculoskeletal reserve [12,53,54]. In this context, PCF may function not only as a measure of respiratory performance but also as an integrated indicator of global neuromuscular capacity and physiological reserve early in recovery [8,55].

BTMs may capture complementary aspects of this systemic milieu, such as metabolic stress driven by inflammation and inactivity [28,39,56]. Beyond reflecting bone resorption, CTX is sensitive to inflammatory signaling and disuse, both of which are prominent in the early post-stroke period [28,57]. Several clinical conditions characterized by frailty or catabolic stress show parallel declines in muscle performance and alterations in bone remodeling, supporting the emerging concept of bone and muscle as an integrated functional unit [31,34]. From this perspective, elevated CTX may serve as a proxy for increased systemic catabolic stress and reduced mechanical loading, which may also affect respiratory and trunk muscles and lead to reduced cough flow [39,56,58]. Conversely, P1NP reflects bone formation, which may be less responsive in acute settings or may change on a different time course than resorption markers [39,59]. This difference may account for the weaker associations observed for P1NP. The divergence between CTX and P1NP in our findings supports their biological plausibility and suggests that bone resorption activity may be a more sensitive indicator of acute systemic stress related to functional performance in the early period after stroke [27,60,61].

Multiple secondary outcomes were evaluated without formal multiplicity correction (e.g., Bonferroni or FDR), which increases the risk of type I error. Therefore, the secondary outcome findings should be interpreted as exploratory and hypothesis-generating, with emphasis on effect sizes and confidence intervals rather than dichotomous statistical significance.

Several limitations should be considered when interpreting these results. First, the retrospective design limits causal inference, and although major conditions and treatments expected to substantially influence cough performance or bone turnover independent of stroke were excluded, residual confounding may persist. BTM levels and respiratory function may have been influenced by unmeasured or incompletely captured factors, such as systemic inflammatory burden, pre-stroke activity level, nutritional status, and baseline frailty. Second, although a standardized PCF protocol was used (three trials with the highest value recorded), performance may still be affected by patient cooperation, cognitive status, fatigue, and technique. Third, pneumonia and suction outcomes were ascertained from routine clinical records, and the operational definition of aspiration pneumonia based on imaging findings plus antibiotic initiation may allow for some misclassification. Fourth, the assessment of multiple secondary outcomes increases the risk of chance findings, and the number of events for some outcomes was limited despite parsimonious adjustment. These limitations underscore the need for prospective validation and replication before drawing firm conclusions regarding prognostic value. Future studies could address these issues through prospective, multicenter designs with standardized timing of BTM and PCF measurements, inclusion of structured dysphagia assessments and respiratory muscle strength measures, and more comprehensive characterization of comorbidities and medication exposure. Longitudinal assessment would be particularly informative to determine whether elevated CTX is associated with persistent cough dysfunction or reflects a transient early state that improves with mobilization and rehabilitation, including pulmonary rehabilitation programs that have been associated with improved respiratory function and diaphragmatic mechanics in other populations [62,63]. Furthermore, if clinical prediction is the goal, future research should evaluate whether CTX adds incremental value beyond established clinical predictors, assess model calibration and discrimination, and perform internal validation.

## 5. Conclusions

Higher CTX levels were independently associated with lower peak cough flow in this retrospective cohort of subacute ischemic stroke patients, whereas associations with P1NP were weaker. In exploratory secondary analyses, higher CTX was also associated with selected respiratory-related outcomes and longer length of stay. These findings are hypothesis-generating and warrant confirmation in prospective multicenter studies that assess reproducibility and incremental predictive value beyond established clinical predictors before any clinical implementation is considered.

## Figures and Tables

**Table 1:** Baseline demographic and clinical characteristics of the patients.

Characteristics	Mean ± SD or n (%)	Median (IQR)
age (years)	63.7 ± 14.1	63.0 (53.0–76.2)
BMI (kg/m^2^)	24.5 ± 3.8	24.9 (21.2–26.8)
onset-to-admission interval (days)	13.8 ± 4.4	14.0 (10.0–18.0)
NIHSS (score)	13.7 ± 6.5	14.0 (8.0–20.0)
K-MBI (score)	49.4 ± 24.1	47.0 (29.8–67.5)
MMSE (score)	18.6 ± 7.2	21.0 (12.0–24.2)
CTX (ng/mL)	0.55 ± 0.26	0.545 (0.357–0.732)
P1NP (ng/mL)	54.25 ± 23.56	56.5 (34.35–72.50)
calcium (mg/dL)	9.20 ± 0.35	9.20 (9.00–9.40)
phosphate (mg/dL)	3.45 ± 0.55	3.50 (3.10–3.90)
ALP (IU/L)	85.7 ± 24.0	88.0 (68.8–102.0)
creatinine (mg/dL)	0.86 ± 0.24	0.86 (0.67–1.00)
eGFR (mL/min/1.73 m^2^)	95.1 ± 17.7	94.4 (82.0–111.2)
hemoglobin (g/dL)	12.76 ± 1.74	13.00 (11.47–13.93)
peak cough flow (L/min)	237.0 ± 85.2	224.0 (174.00–283.25)
length of stay (days)	47.2 ± 19.3	35.0 (31.0–68.0)
suction count (times)	8.6 ± 6.7	7.0 (2.8–14.2)
sex: male	69 (61.6%)	—
sex: female	43 (38.4%)	—
smoking	39 (34.8%)	—
non-smoking	73 (65.2%)	—
brainstem lesion	30 (26.8%)	—
non-brainstem lesion	82 (73.2%)	—
low PCF (<160 L/min)	25 (22.3%)	—
aspiration pneumonia	26 (23.2%)	—

Abbreviations: ALP, alkaline phosphatase; BMI, body mass index; CTX, C-terminal telopeptide of type I collagen; eGFR, estimated glomerular filtration rate; Hb, hemoglobin; K-MBI, Korean version of the Modified Barthel Index; MMSE, Mini-Mental State Examination; NIHSS, National Institutes of Health Stroke Scale; PCF, peak cough flow; P1NP, procollagen type 1 N-terminal propeptide.

**Table 2:** Correlation between bone turnover markers and peak cough flow.

Pair	Correlation Coefficient	*p*-Value
CTX (ng/mL) vs. PCF (L/min)	−0.469	<0.001
P1NP (ng/mL) vs. PCF (L/min)	−0.213	0.024
CTX (ng/mL) vs. PCF (L/min), adjusted for NIHSS	−0.494	<0.001

Abbreviations: CTX, C-terminal telopeptide of type I collagen; PCF, peak cough flow. Note: Unadjusted associations are shown as Spearman rho; the NIHSS-adjusted CTX–PCF association is shown as a partial Spearman correlation coefficient.

**Table 3:** Multivariable linear regression models of peak cough flow according to bone turnover markers.

Variable	CTX Model β (95% CI)	*p*	P1NP Model β (95% CI)	*p*	CTX Model Standardized β	P1NP Model Standardized β
CTX (per 1 SD increase)	−42.32 (−56.12, −28.52)	<0.001	—	—	−0.499	—
P1NP (per 1 SD increase)	—	—	−18.33 (−34.28, −2.38)	0.025	—	−0.216
age (years)	−0.98 (−1.96, 0.00)	0.05	−0.89 (−2.01, 0.22)	0.116	−0.162	−0.148
male sex (vs female)	13.70 (−14.31, 41.71)	0.334	12.28 (−20.04, 44.60)	0.453	0.079	0.07
BMI (kg/m^2^)	1.58 (−2.16, 5.31)	0.404	2.31 (−1.94, 6.56)	0.284	0.07	0.102
onset-to-admission interval (days)	−1.17 (−4.34, 1.99)	0.464	−1.95 (−5.55, 1.66)	0.286	−0.06	−0.1
NIHSS (score)	−3.27 (−5.40, −1.13)	0.003	−2.48 (−4.94, −0.03)	0.048	−0.249	−0.189
K-MBI (score)	0.22 (−0.35, 0.80)	0.444	0.04 (−0.62, 0.69)	0.911	0.063	0.01
eGFR (mL/min/1.73 m^2^)	−0.04 (−0.81, 0.72)	0.913	−0.27 (−1.14, 0.60)	0.539	−0.009	−0.056
smoking (vs non-smoking)	−1.11 (−29.60, 27.39)	0.939	−13.12 (−45.87, 19.63)	0.429	−0.006	−0.074
brainstem lesion (vs non-brainstem lesion)	1.08 (−31.15, 33.30)	0.947	−15.24 (−51.44, 20.96)	0.406	0.006	−0.08

Adjusted R^2^: CTX model = 0.294; P1NP model = 0.082. Cohen’s f^2^: CTX model = 0.37; P1NP model = 0.05. Abbreviations: β, regression coefficient; BMI, body mass index; CI, confidence interval; CTX, C-terminal telopeptide of type I collagen; eGFR, estimated glomerular filtration rate; K-MBI, Korean version of the Modified Barthel Index; NIHSS, National Institutes of Health Stroke Scale; P1NP, procollagen type 1 N-terminal propeptide; SD, standard deviation. Note: BTM effects are per 1 SD increase (z-score). Covariates included (when available): age (years), male sex (vs. female), BMI (kg/m^2^), onset-to-admission interval (days), NIHSS (score), K-MBI (score), eGFR (mL/min/1.73 m^2^), smoking (vs. non-smoking), and brainstem lesion (vs. non-brainstem lesion).

**Table 4:** Associations of bone turnover markers with secondary outcomes in adjusted regression models.

Outcome	Predictor (per 1 SD Increase)	Effect	95% CI	*p*
low PCF (PCF < 160 L/min)	CTX	OR 2.93	1.59–5.40	<0.001
	P1NP	OR 1.47	0.89–2.42	0.133
aspiration pneumonia	CTX	OR 1.70	1.02–2.81	0.040
	P1NP	OR 1.04	0.64–1.69	0.859
length of stay (log(LOS))	CTX	9.0%	1.7–16.9%	0.017
	P1NP	0.3%	–6.8% to 7.9%	0.946
suction count	CTX	IRR 1.17	0.97–1.42	0.104
	P1NP	IRR 0.96	0.80–1.15	0.649

Abbreviations: CI, confidence interval; CTX, C-terminal telopeptide of type I collagen; IRR, incidence rate ratio; LOS, length of stay; OR, odds ratio; PCF, peak cough flow; P1NP, procollagen type 1 N-terminal propeptide; SD, standard deviation. Note: Logistic regression was used for low PCF and aspiration pneumonia (effect reported as OR). LOS was modeled with linear regression on log(LOS) (effect reported as % change). Suction count was modeled using negative binomial regression (effect reported as IRR). Parsimonious covariates included (when available): age (years), male sex (vs female), NIHSS (score), eGFR (mL/min/1.73 m^2^), smoking (vs non-smoking), and brainstem lesion status.

## Data Availability

The datasets generated and/or analyzed in the current study are available from the corresponding author upon reasonable request.
